# Linear Malignant Melanoma In Situ: Reports and Review of Cutaneous Malignancies Presenting as Linear Skin Cancer

**DOI:** 10.7759/cureus.1696

**Published:** 2017-09-18

**Authors:** Philip R Cohen

**Affiliations:** 1 Department of Dermatology, University of California, San Diego

**Keywords:** cancer, cutaneous, in situ, linear, malignant, malignant melanoma, melanoma in situ, neoplasm, skin

## Abstract

Melanomas usually present as oval lesions in which the borders may be irregular. Other morphological features of melanoma include clinical asymmetry, variable color, diameter greater than 6 mm and evolving lesions. Two males whose melanoma in situ presented as linear skin lesions are described and cutaneous malignancies that may appear linear in morphology are summarized in this report. A medical literature search engine, PubMed, was used to search the following terms: cancer, cutaneous, in situ, linear, malignant, malignant melanoma, melanoma in situ, neoplasm, and skin. The 25 papers that were generated by the search and their references, were reviewed; 10 papers were selected for inclusion. The cancer of the skin typically presents as round lesions. However, basal cell carcinoma and squamous cell carcinoma may arise from primary skin conditions or benign skin neoplasms such as linear epidermal nevus and linear porokeratosis. In addition, linear tumors such as basal cell carcinoma can occur. The development of linear cutaneous neoplasms may occur secondary to skin tension line or embryonal growth patterns (as reflected by the lines of Langer and lines of Blaschko) or exogenous factors such as prior radiation therapy. Cutaneous neoplasms and specifically melanoma in situ can be added to the list of linear skin lesions.

## Introduction

Malignant cutaneous neoplasms of the skin include basal cell carcinoma, squamous cell carcinoma, and melanoma. Typically, skin cancers present as oval tumors [1]. The clinical appearance of skin cancers in a linear presentation is uncommon. Two males whose melanoma in situ presented as linear tumors is described. In addition, other skin malignancies such as basal cell carcinoma and squamous cell carcinoma with a linear morphology are summarized.

## Case presentation

Case one

A 72-year-old male with a history of malignant melanoma on his upper back (Breslow depth 0.33 mm, Clark level II), nine years earlier presented with a new pigmented lesion on his left chest. He had no prior trauma or radiation to the site.

**Figure 1:**
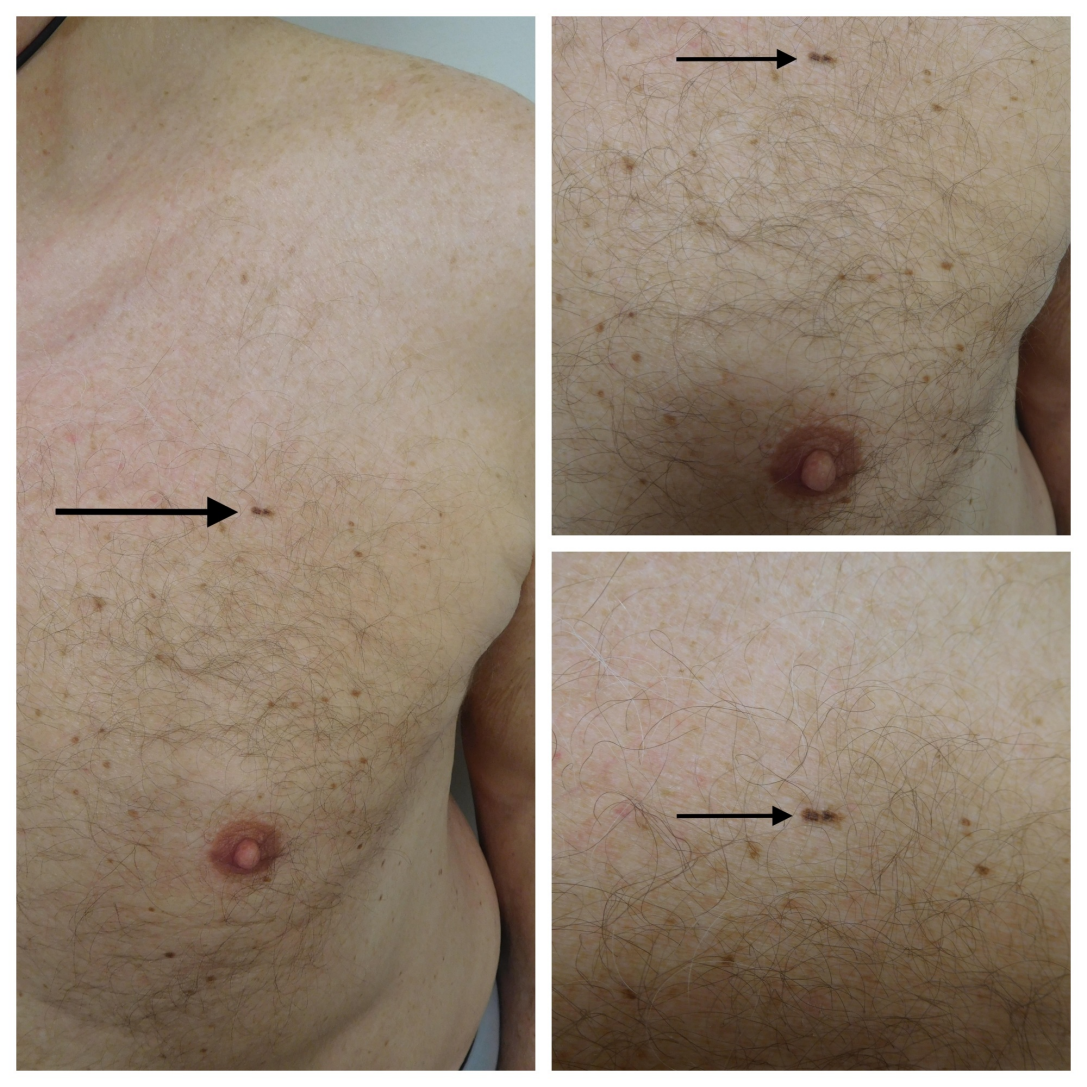
Distant (left) and closer (upper right and lower right) views of the clinical presentation of the linear melanoma in situ on the left chest of a 72-year-old male. The linear tumor (solid arrow) is horizontally positioned above the breast. The lateral sides are hyperpigmented and there is a small area of hypopigmentation in the center of the tumor. The melanoma in situ measures 8 x 2 mm with a resultant length-to-width ratio of 4:1.

Cutaneous examination showed a linear pigmented patch that was located horizontally on his left upper chest superior to the breast. The lesion measured 8 x 2 mm. Both of the distal ends were dark brown and there was a 1 mm hypopigmented area in the center (Figure 1).

**Figure 2:**
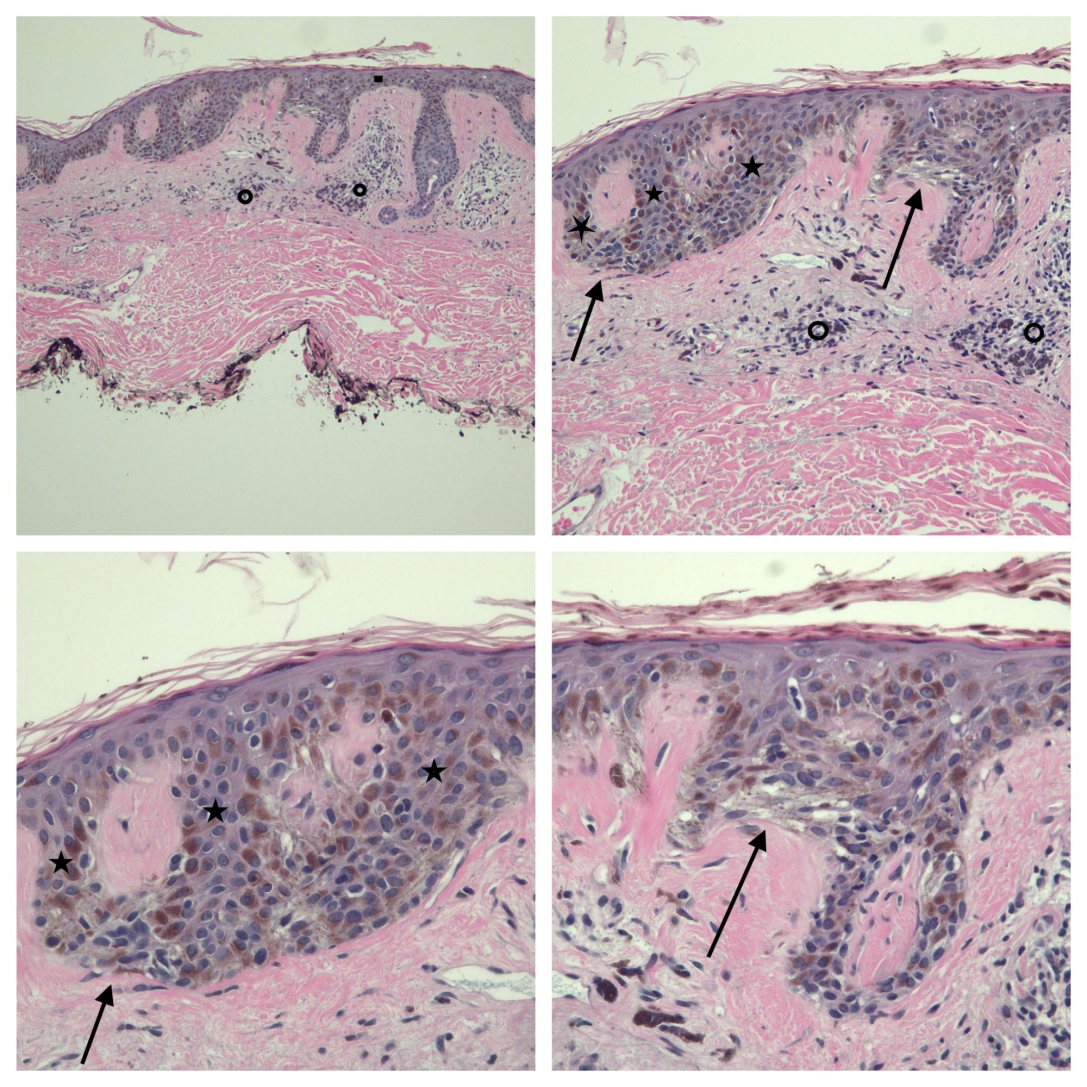
Low (upper left), intermediate (upper right), and higher (lower left and lower right) magnification views of the pathology of the linear melanoma in situ from the left chest of a 72-year-old male. There are compact orthokeratosis and focal parakeratosis; the epithelium (solid rectangle) is thinned. There is the elongation of the rete ridges (solid stars) and anastomosis of the tips of the rete ridges. Melanocyte nests (solid arrows) of variable size and shape are present in the lower layers of the epidermis. Lymphocytes and melanophages are present around the vessels in the papillary dermis (open circles). There is no invasion of the tumor melanocytes into the dermis (hematoxylin and eosin (HE) stain; upper left, x 4, upper right, x 10; lower left, x 20 and lower right, x 20).

A shave biopsy that removed the entire lesion was performed. Microscopic evaluation showed a broad, poorly circumscribed, intraepithelial proliferation of melanocytes with atypical nuclei overlying sun-damaged dermis. The melanocytes were arranged in nests of varying sizes and shapes; in addition, the melanocyte nests are distributed in a haphazard pattern along the epidermal-dermal junction. There was no invasion of the atypical melanocytes into the dermis (Figure 2).

Correlation of the clinical presentation and the pathological findings established a diagnosis of linear melanoma in situ. The lesion site was excised with 5 mm margins of normal skin and a side-to-side closure of the wound edges was performed. Sequential follow-up examinations at three, six, nine and 12 months showed excellent healing of the surgical site without evidence of tumor recurrence.

Case two

A healthy 31-year-old male presented for an examination of his skin. He was unaware of the pigmented lesions on his back.

**Figure 3:**
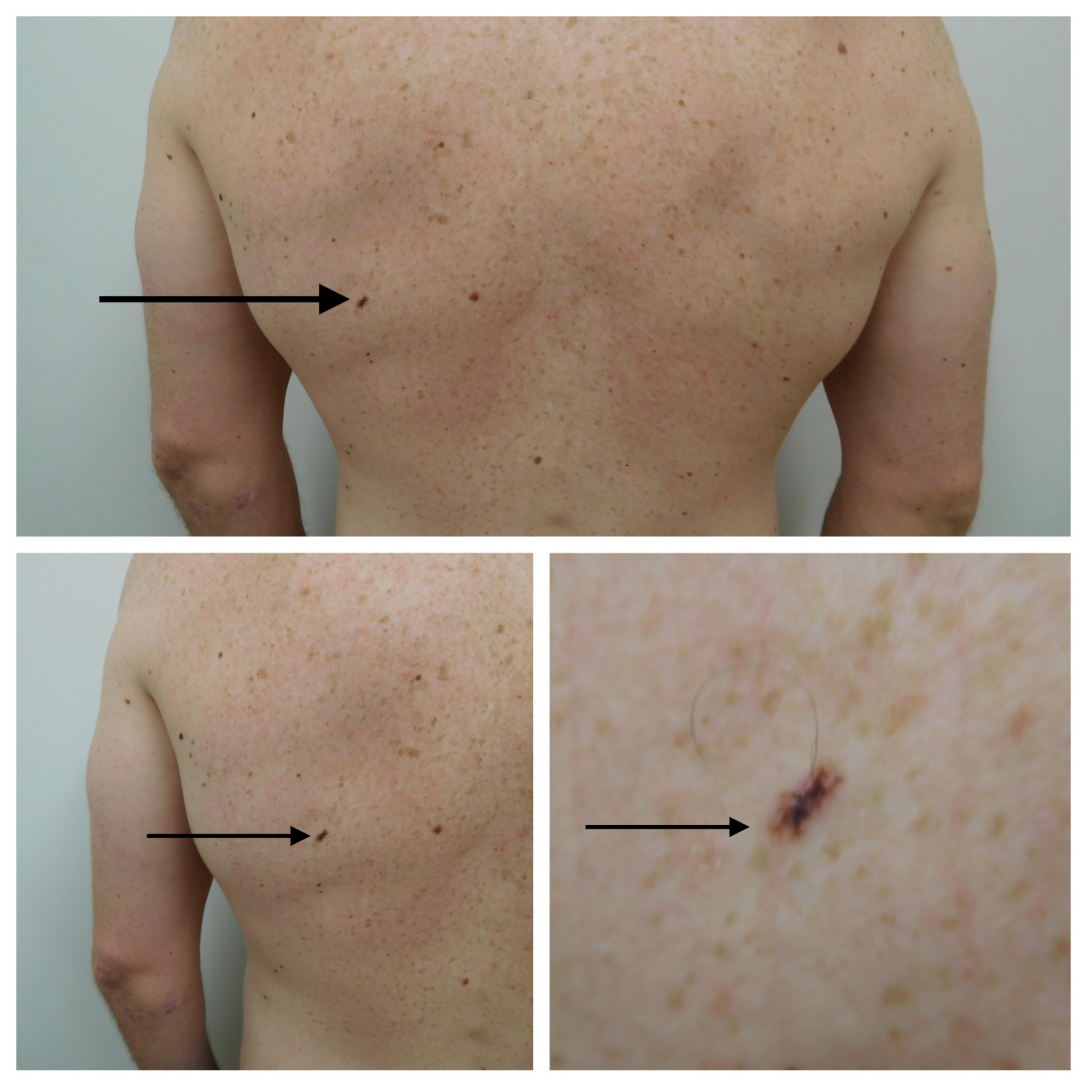
Distant (top) and closer (lower right and lower left) views of the clinical presentation of linear melanoma in situ arising in a compound melanocytic nevus on the left mid-back of a 31-year-old male. In addition to the tumor (solid arrow), numerous lentigos, appearing as brown patches also present on the upper back. The tumor is brown with overlying irregular black pigmentation. The tumor (solid arrow) measures 10 x 2.8 mm with a resultant length-to-width ratio of 3.6:1.

Cutaneous examination showed numerous brown patches, clinically consistent with lentigos, on the upper two-thirds of his back. A linear, brown and dark black, pigmented plaque was located on his left mid-back below the scapula. The lesion measured 10 x 2.8 mm (Figure 3).

A 3-mm punch biopsy was performed. Microscopic evaluation showed a compound melanocytic lesion with biphasic composition. Small ovoid benign-appearing nests of melanocytes were in the dermis. Darkly pigmented melanocytes, present not only as nests but also as an increased number of individual cells and this was present along with the basal layer of the epidermis.

Correlation of the clinical presentation and the pathological findings established a diagnosis of linear melanoma in situ arising in a compound melanocytic nevus. The lesion was excised with a 1 cm margin of normal skin and side-to-side closure of the wound edges was performed. Follow-up examinations at three months showed excellent healing of the surgical site without evidence of tumor recurrence.

## Discussion

Melanoma in situ is a cutaneous malignancy of melanocytes within the epidermis, the melanocytes do not invade into the dermis. Clinical subtypes of in situ melanoma include lentigo maligna, superficial spreading, acral lentiginous, and mucosal. The pathology shows a pagetoid spread of the tumor cells demonstrated by a proliferation of single cells or groups of atypical melanocytes along the basal layer of the epidermis or throughout the epidermis into the granular or horny layers or both [1-2]. The tumors from both the patients in this report had these pathologic features.

The incidence of melanoma in situ has increased by about 9.5% annually over the last 10 years. The median age at diagnosis is 63 years, and the most commonly found among the Whites. The head and neck are the most common sites for melanoma in situ [1].

The clinical features of melanoma in situ and melanoma follow the ABCDEs: asymmetry, border irregularity, color (which is uneven), diameter greater than 6 mm, and evolution (in which the lesion changes over time). The morphology of melanoma in situ is typically oval in appearance [1]. However, the patients' tumors were linear; in addition, their tumors were multicolored and larger than 6 mm in diameter.

Linear cutaneous malignancies are uncommon. Several observations of the primary linear basal cell carcinoma have been described [3-8]. The basal cell carcinoma [8] and squamous cell carcinomas [9-10] have also been observed to develop in benign linear dermatoses and skin neoplasms, such as epidermal nevus [8-9] and porokeratosis [10].

The criteria used to classify a basal cell carcinoma as linear includes a length-to-width ratio of 3:1 or more [4]. The melanoma in situ lesions described in the reported patients had a length-to-width ratio of 4:1 and 3.6:1. To the best of my knowledge, the males in this report are the first patients with linear melanoma in situ to be described in the literature. Although it is possible that additional individuals with primary cutaneous melanomas that presented as linear tumors have been observed, the details of these patients have not been published.

The pathogenesis of linear malignancies remains to be established and may be multifactorial. Linear basal cell carcinoma has been described in skin previously exposed to ionizing radiation; it was speculated that the changes in the dermis following radiation therapy influenced the growth pattern of the tumor that developed in that site [5]. Another possibility is that linear tumors may reflect the skin tension lines in that location. Alternatively, it may be postulated that the tumors align themselves along the lines of Langer or lines of Blaschko [7].

The treatment of linear cutaneous tumors is the same as that performed for similar neoplasms that are oval. 

## Conclusions

Basal cell carcinoma, squamous cell carcinoma, and melanoma in situ are cutaneous neoplasms that may present, albeit rarely, as linear lesions. The development of these tumors with a linear morphology may be multifactorial and include either exogenous factors (such as ionizing radiation), the influence of the skin tension lines, or embryonal growth development patterns (such as lines of Langer and lines of Blaschko). Linear pigmented lesions can be added to the differential diagnosis of melanoma in situ. In addition, melanoma in situ should be considered in the differential diagnosis of cutaneous linear skin lesions.
